# Tumor resection at the pelvis using three-dimensional planning and patient-specific instruments: a case series

**DOI:** 10.1186/s12957-016-1006-2

**Published:** 2016-09-21

**Authors:** Thorsten Jentzsch, Lazaros Vlachopoulos, Philipp Fürnstahl, Daniel A. Müller, Bruno Fuchs

**Affiliations:** 1 Department of Orthopedics, Balgrist University Hospital, University of Zurich, Forchstrasse 340, 8008 Zurich, Switzerland; 2 Computer Assisted Research and Development (CARD) Group, Balgrist University Hospital, University of Zurich, Zurich, Switzerland

**Keywords:** Sarcomas, Pelvis, Three-dimensional (3-D, 3D) planning, 3-D-printed models, Patient-specific instruments (PSI), Patient-specific guides (PSG), Patient-specific templates (PST), Resection margins

## Abstract

**Background:**

Sarcomas are associated with a relatively high local recurrence rate of around 30 % in the pelvis. Inadequate surgical margins are the most important reason. However, obtaining adequate margins is particularly difficult in this anatomically demanding region. Recently, three-dimensional (3-D) planning, printed models, and patient-specific instruments (PSI) with cutting blocks have been introduced to improve the precision during surgical tumor resection. This case series illustrates these modern 3-D tools in pelvic tumor surgery.

**Methods:**

The first consecutive patients with 3-D-planned tumor resection around the pelvis were included in this retrospective study at a University Hospital in 2015. Detailed information about the clinical presentation, imaging techniques, preoperative planning, intraoperative surgical procedures, and postoperative evaluation is provided for each case. The primary outcome was tumor-free resection margins as assessed by a postoperative computed tomography (CT) scan of the specimen. The secondary outcomes were precision of preoperative planning and complications.

**Results:**

Four patients with pelvic sarcomas were included in this study. The mean follow-up was 7.8 (range, 6.0–9.0) months. The combined use of preoperative planning with 3-D techniques, 3-D-printed models, and PSI for osteotomies led to higher precision (maximal (max) error of 0.4 centimeters (cm)) than conventional 3-D planning and freehand osteotomies (max error of 2.8 cm). Tumor-free margins were obtained where measurable (*n* = 3; margins were not assessable in a patient with curettage). Two insufficiency fractures were noted postoperatively.

**Conclusions:**

Three-dimensional planning as well as the intraoperative use of 3-D-printed models and PSI are valuable for complex sarcoma resection at the pelvis. Three-dimensionally printed models of the patient anatomy may help visualization and precision. PSI with cutting blocks help perform very precise osteotomies for adequate resection margins.

## Background

Sarcomas account for more than 20 % and less than 1 % of solid malignancies in children and adults, respectively [1]. There are approximately 15,000 new cases per year in the USA [2]. Sarcomas are heterogeneous malignant mesenchymal neoplasms of the soft tissues, visceral organs, and bones. Five-year survival rates for some of the most common primary nonmetastatic sarcomas of the bone, osteosarcoma and chondrosarcoma, range around 70 and 50 %, respectively. If located in the axial skeleton, however, they substantially decrease to 35 and 47 %, respectively. At the pelvis, sarcomas are associated with rather high local recurrence rates of around 30 % [3]. In this anatomically demanding region, this is partially attributed to the challenge of obtaining adequate margins, which plays a major role for local recurrence. In a previous case series of 18 patients [4], local recurrence occurred in 33 % of patients with intralesional or marginal margins, but only in 11 % with wide margins. Due to the complex three-dimensional (3-D) geometry of the pelvis and close proximity to vital structures, safe margins may not be reached at all times in state-of-the-art limb salvage surgeries; especially at the sciatic notch and SI joint. Therefore, surgical approaches require meticulous preoperative planning and trained skills. However, even experienced surgeons may not accurately reach adequate margins at all times [5].

Navigation systems have assisted intraoperative guidance and led to a reduction of intralesional resection rates [6]. Recent advances have also seen 3-D-printed models of the patient anatomy and patient-specific instruments (PSI) with cutting blocks functioning as drill or saw-guides generated based on 3-D preoperative planning. Originally designed for oral and maxillofacial surgery [7] and previously used for total knee arthroplasties [8, 9], the use of PSI has broadened to many other body parts [10–12], including the pelvis [13]. It also offers greater precision for the resection of bone tumors [14]. A recent study by Gouin et al. [15] reported that location accuracy averaged 2.5 millimeters (mm) in nine cut planes of seven patients out of a case series of 11 patients with pelvic bone tumors treated with PSI.

The presented case series of four patients illustrates varying complexity levels of 3-D planning; from conventional freehand osteotomies over printout models to PSI using cutting blocks. The ultimate goals are to improve the precision of osteotomies for more accurate tumor margins and to prevent local recurrences.

## Methods

The local ethical committee issued a waiver (Zurich Cantonal Ethics Commission, KEK-ZH 80-2015) for this case series, and all patients gave their informed consent for their participation in and publication of this study. All patients with a 3-D-planned pelvic tumor resection, performed at the author’s institution in 2015, were included in this study (*n* = 4). The primary outcome variable was tumor-free resection margins (Table 1). The secondary outcome variables were the precision of the osteotomy with respect to the preoperative planning as well as complications. Local recurrence and metastasis at last follow-up were also recorded.

**Table 1:** Patient characteristics (*n* = 4)

Case	Age (y)	Sex	Tumor	Location	Surgery	Chemotherapy	Follow-up (mths)	Planning	Osteotomy	Tumor-free margin	Metastasis	Precision (max error (cm))	Complication
1	14	F	Ewing sarcoma	Ilium	Resection	Yes^a^	6	3-D	Freehand	Yes	No	2.8	No
2	42	M	Giant cell tumor	SI joint	Resection and curettage	No	9	Print-out	Freehand	NA^b^	No	NA^b^	Yes^c^
3	53	M	Chondrosarcoma	Gluteus maximus	Resection	No	7	Print-out	Freehand	Yes	No	NA^b^	Yes^d^
4	51	M	Chondrosarcoma	Ilium and hip joint	Resection	No	9	3-D and print-out	PSI	Yes	No	0.4	Yes^c^

*y* years, *mths* months, *max* maximal, *cm* centimeters, *F* female, *M* male, *3-D* three-dimensional, *SI* sacroiliac, *NA* not applicable, *PSI* patient-specific instrument

^a^Neoadjuvant and adjuvant

^b^Due to curettage. Subjectively better precision than 3-D planning and worse precision than PSI

^c^Asymptomatic insufficiency fracture of the inferior pubic ramus

^d^Subacute, ischemic infarction of the left cuneus with severe headaches and homonymous hemianopsia

**Fig. 1:**
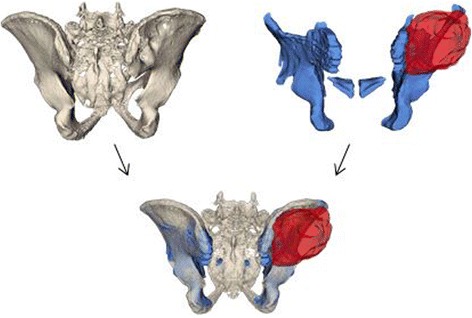
Superimposition of CT (*upper left*; *beige*) and MRI (*upper right*; *blue*) images to generate a 3-D model (*bottom*) of the tumor (*red*) and its surrounding structures

The first step of the preoperative planning consisted in the generation of 3-D triangular surface models of the pelvis from computed tomography (CT) (Philips Brilliance 40 CT, Philips Healthcare, The Netherlands) and magnetic resonance imaging (MRI) scans (3.0 T Skyra-fit, Avanto-fit, Siemens Healthcare, Erlangen, Germany, with the contrast agent gadobutrol (Gadovist, Bayer Schering Pharma, Germany)). The models were generated using Mimics software (Materialise, Belgium). In a next step, the CT-reconstructed bone and the MRI-reconstructed tumor were fused by using the preoperative planning software application (CASPA, Balgrist CARD AG, Zurich, Switzerland). Fusing CT and MRI data was useful in order to display osseous structures with generally higher resolution of CTs and soft tissues with a generally lower resolution of MRIs (Fig. 1). Planning of the resection and designing of the PSI was performed by an experienced surgeon using the CASPA software.

Postoperative assessment was done by acquiring a CT scan of the resected tumor specimen. Then, 3-D models were generated and superimposed with the preoperative bone models to quantify the accuracy of the resection with respect to the preoperatively planned osteotomy planes.

### Case 1

**Fig. 2:**
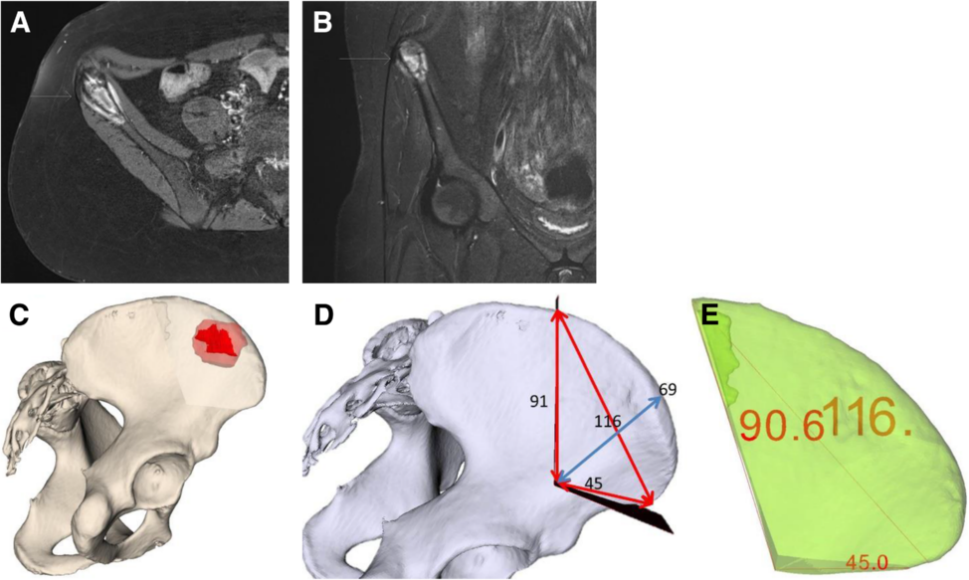
Case 1: preoperative planning. MRI of a Ewing sarcoma (*arrows*) at the right ilium (2-A-B) and planning (2-C-E) with computer-assisted research and development (CARD) before (2-A-B) and after (2-C-E) neoadjuvant chemotherapy. **a** T1 axial MRI plane. **b** T2 turbo inversion recovery magnitude (TIRM) coronal MRI plane. **c** Ewing sarcoma (*red*) at the right ilium (*beige*). **d** 3-D-planned measurements (mm) of osteotomy planes. **e** Preoperative 3-D-planned tumor resection specimen (mm) (*green*)

A 14-year-old otherwise healthy female complained of pain in her right pelvis for 1 month during her first medical consultation. After being nonresponsive to analgesics, an X-ray was obtained. Due to a suspicious osteolytic lesion in the right ilium, a MRI was ordered (Fig. 2). The patient was referred to another hospital, and the diagnosis of Ewing sarcoma of the right ilium with a *t*(11;22) translocation (EWSR1:FLI1 fusion protein) was confirmed with a biopsy. Tumor staging using positron emission tomography-computed tomography (PET-CT) did not reveal any metastases. This was followed by implantation of a port and ovarian tissue cryopreservation. Neoadjuvant chemotherapy with vincristine, ifosfamide, doxorubicin, and etoposide was initiated according to the VIDE regimen and EWING 2008 protocol [16] and lasted for 4 months.

Preoperative planning consisted of 3-D model generation of the bone and tumor and the definition of the osteotomy planes (Fig. 2). Measurements for the entry points and course of the osteotomy planes were taken. However, no 3-D-printed bone model or PSI were available, and osteotomies were carried out freehandedly.

**Fig. 3:**
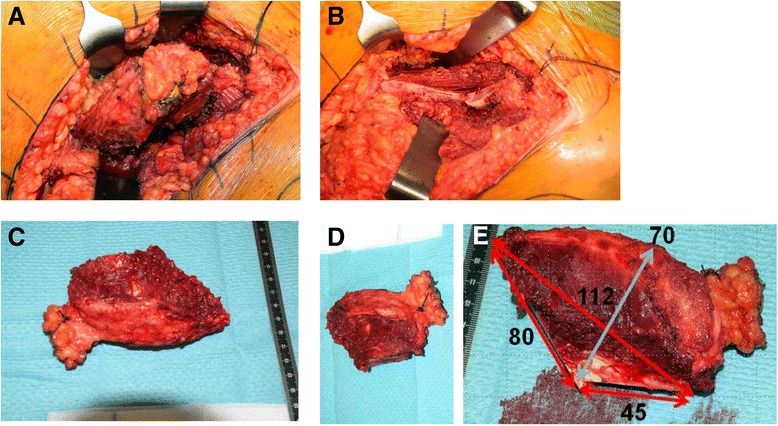
Case 1: intraoperative findings. **a** Tumor at the ilium. **b** Ilium after tumor resection. **c** Extra-pelvic side of the resected tumor specimen. **d** Intrapelvic side of the resected tumor specimen. **e** Intraoperative measurements (millimeters) of the resected tumor specimen

**Fig. 4:**
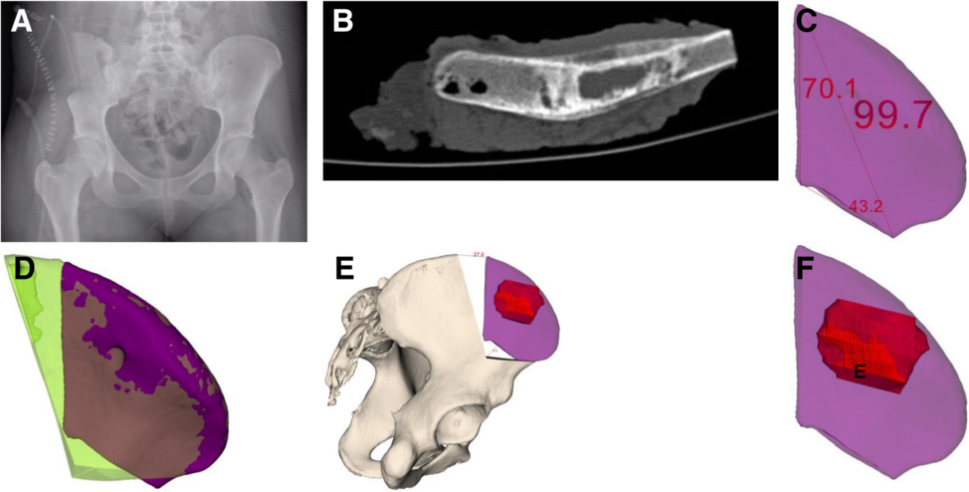
Case 1: postoperative evaluation. X-ray (4-A), CT (4-B), and comparison of 3-D-planned (*green)* and actually resected (*purple*) tumor specimens (4-C-F). **a** Postoperative antero-posterior X-ray of the pelvis. **b** Axial computed tomography (CT). **c** Postoperative 3-D evaluation of tumor resection specimen (mm). **d** Comparison of planned and actual tumor resection specimens. **e** Orientation (mm) of actual tumor resection specimen to the pelvis without planned resected tumor specimen (*white*). **f** Tumor (*red*) orientation within the resected tumor specimen. **e** Orientation (mm) of actual tumor resection specimen to the pelvis without planned resected tumor specimen (*white*). **f** Tumor (*red*) orientation within the resected tumor specimen

The surgical resection of the right anterior supra-acetabular ilium was performed three weeks after the end of preoperative chemotherapy. Using an extended iliofemoral approach, the abdominal wall including the inguinal ligament and the iliacus, gluteal, sartorius, and tensor fasciae latae muscles were detached. After adequate visualization of the planned osteotomy planes, measurements were taken with a ruler to confirm that the entry points were as planned preoperatively. The supra-acetabular transition point was marked with a Kirschner wire. Freehand osteotomies aimed at the Kirschner wire and started below the anterior superior iliac spine (ASIS) anteriorly and 11.6 centimeters (cm) away from the ASIS posteriorly. The resulting specimen was removed en bloc (Fig. 3). The ASIS was reconstructed with transosseous refixation of the inguinal ligament and sartorius muscle—attached to an ASIS bone block—before layered closure of the abdominal wall and skin. An X-ray of the pelvis was acquired in the immediate postoperative period (Fig. 4). The postoperative rehabilitation included ambulation as tolerated, starting at the first postoperative day.

The histopathological analysis of the resected specimen reported minimal resection margins of 0.8 cm within the bone, 0.4 cm intrapelvically, 0.6 cm extra-pelvically, 0.9 cm anteriorly, and 1.5 cm posteriorly. A postoperative CT scan of the resected specimen was performed and compared to the preoperative planning (Fig. 4).

At the last follow-up, 6 months postoperatively, the patient had just finished the last adjuvant chemotherapy cycle. She reported an uneventful course without substantial pain or functional deficit. On clinical evaluation, the initially mildly hypertrophic scar was normal, no severe pain to palpation was noted, the hip flexors and adductors were mildly decreased (muscular strength (M) 4 out of 5) with a mildly positive Trendelenburg’s sign, and a small perifocal hypoesthesia directly inferior to the scar at the anterolateral thigh was present without any significant neurological deficits. The MRI showed regular postoperative changes. The patient had started a professional trainee program in the meantime.

### Case 2

**Fig. 5:**
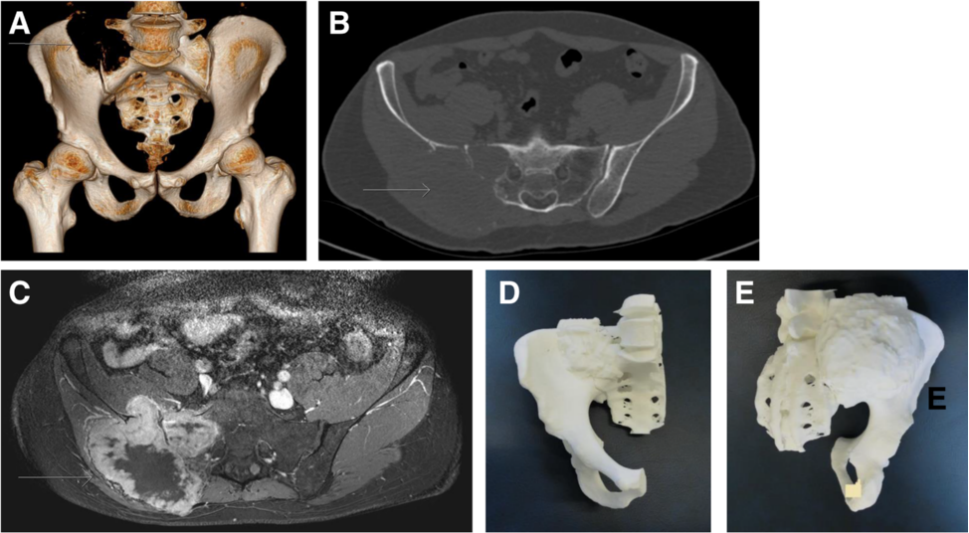
Case 2: preoperative planning. CT (5-A-B) and MRI (5-C) of a giant cell tumor (*arrows*) affecting the iliosacral joint and in close proximity to the first sacral nerve root as well as preoperative planning (5-D-E) with a three-dimensionally printed models of the right hemipelvis including the sacrum and the tumor. **a** Three-dimensional (3-D) reconstruction of the pelvis. **b** Axial plane of the CT showing the osteolytic lesion. **c** T1 contrast-enhanced axial MRI plane. **d** Anterior view of the 3-D printed model. **e** Posterior view of the 3-D printed model

A 42-year-old male without any secondary diseases presented with pain at the lower back and right pelvis. The pain started 2 years earlier, and an X-ray at an external institution did not show any specific lesions. After a spontaneous pain-free period of one and a half years, the pain recurred. Despite mild improvements with physiotherapy and osteopathy, a CT scan and MRI were obtained. These displayed a large soft tissue mass in the right pelvis, an osteolytic lesion of the lateral mass of the sacrum, a cranially affected iliosacral joint, and close proximities to the first sacral (S1) nerve root as well as the posterior skin (Fig. 5). The following ultrasound-guided biopsy showed a giant cell tumor. To decrease the sampling error and exclude the differential diagnosis of a giant cell-rich osteosarcoma, a CT-guided biopsy was performed two weeks later, which confirmed the histopathological analysis. No metastasis was found in the CT scan of the thorax.

For a more precise visualization, a 3-D pelvic model was generated according to the CT scan and manufactured (by Medacta SA; Castel St. Pietro, Switzerland) using a selective laser sintering device. Biocompatible polyamide 12 (i.e., PA2200) was used as raw material. Conventional steam pressure was applied for sterilization. Preoperatively, an angiography and embolization were performed to decrease the risk of massive intraoperative bleeding. The extra-osseous part was resected (Fig. 5), while curettage was chosen for the intraosseous part close to the S1 nerve root. The latter was chosen despite a small osseous bridge above the sciatic notch because the patient was nearly pain-free indicating that the osteolysis did not affect mechanical stability. Furthermore, no reconstruction eases postoperative follow-up for better monitoring of potential local recurrence.

A dorsal iliac approach was chosen. The gluteus maximus muscle was detached under visualization of the unaffected inferior gluteal nerve. The extra-pelvic part of the tumor was visualized and resected beginning at the iliac crest. Despite the preoperative embolization, several large vessels had to be coagulated. Intrapelvic curettage was performed along the sciatic notch, lateral mass of the sacrum and medial iliac bone, where the iliacus fascia served as a tumor barrier. Then, the iliosacral joint was identified and the cartilage removed. Advancing further medially with the curettage, the S1 nerve root was identified visually and, thus, left intact. After complete tumor removal, curettage, and bleeding control, the gluteal muscles were reattached and the skin closed (Fig. 5). The postoperative protocol allowed weight-bearing as tolerated starting at the first postoperative day. After an interdisciplinary tumor board meeting, the patient was prescribed denosumab to potentially decrease the risk of local recurrence.

Seven months postoperatively, the patient was pain-free without any functional deficit, even when playing golf without restrictions. The MRI did not show any signs of local recurrence. Therefore, denosumab was stopped. However, 8 months postoperatively, the patient complained of a traumatic pain with fast ambulation at the left hip. He did not report any pain at rest or with slow ambulation. Although X-rays did not show a fracture, MRI revealed a non-displaced insufficiency fracture of the contralateral left superior pubic ramus. This fracture was attributed to the altered mechanics after the tumor removal. Therefore, the patient was still ambulating with limited touch-ground weight-bearing at the last follow-up 9 months postoperatively.

### Case 3

**Fig. 6:**
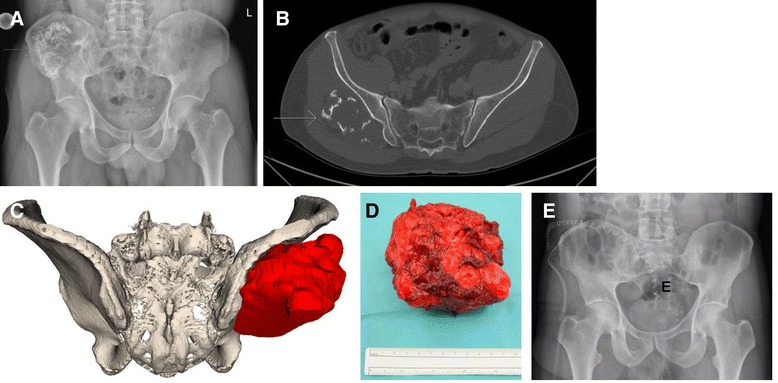
Case 3: preoperative planning, intraoperative findings, and postoperative evaluation of an extra-pelvic chondrosarcoma of the right ilium (*arrows*). **a** X-ray of the pelvis. **b** Axial plane of a CT scan revealing an extra-pelvic location of the tumor only affecting the external pelvic cortex. **c** Three-dimensionally printed model of the tumor for the preoperative planning. **d** Resected tumor specimen. **e** Postoperative antero-posterior X-ray

A 53-year-old male with no concomitant disease observed a hard, painless mass at the pelvis for about four months. He consulted his general practitioner, who referred him to a regional hospital. The X-ray and CT scan showed a large gluteal chondrogenic mass originating from the right posterior iliac wing (Fig. 6). The lesion did not affect the iliosacral joint or sciatic nerve. A CT-guided biopsy revealed nonspecific chondrogenic tissue. The patient was referred to our hospital for further evaluation. After review of the patient charts, a low-grade chondrosarcoma, most likely secondary to previous unknown osteochondroma, was found. No metastasis was present in the subsequent staging examinations.

The origin of the lesion was close the posterior superior iliac spine (PSIS). The ilium itself was not invaded by the tumor. For better stability of the pelvic ring, tumor excision was planned at its base at the PSIS, preserving the tabula interna of the ilium and the sacroiliac joint. The pelvis including the tumor was printed out as a 3-D model for detailed preoperative planning of the freehand osteotomy (Fig. 6).

During surgery, the patient was placed in a lateral position and the skin incision started at the distal end of the sacrum, following the PSIS and the iliac crest up to the ASIS. The gluteal muscles were dissected from the iliac bone from proximally to distally, preserving the superior gluteal neurovascular bundle. As planned on the 3-D model, the chisel was guided from below the PSIS posteriorly to between the iliac cortices anteriorly. The tumor was resected en bloc (Fig. 6), and the gluteal muscles were reattached.

The postoperative histologic work up revealed a low-grade chondrosarcoma (grade (G) 1), resected with adequate margins. Since most of the resected specimen included soft tissues and the resection margins were not specifically calculated preoperatively, no precision was calculated using a postoperative CT scan. The patient was mobilized with crutches and full weight-bearing. At the last follow-up, 7 months postoperatively, the patient was able to ambulate without crutches and without a limp. In the MRI of the pelvis and CT of the chest, no further disease was detected.

### Case 4

**Fig. 7:**
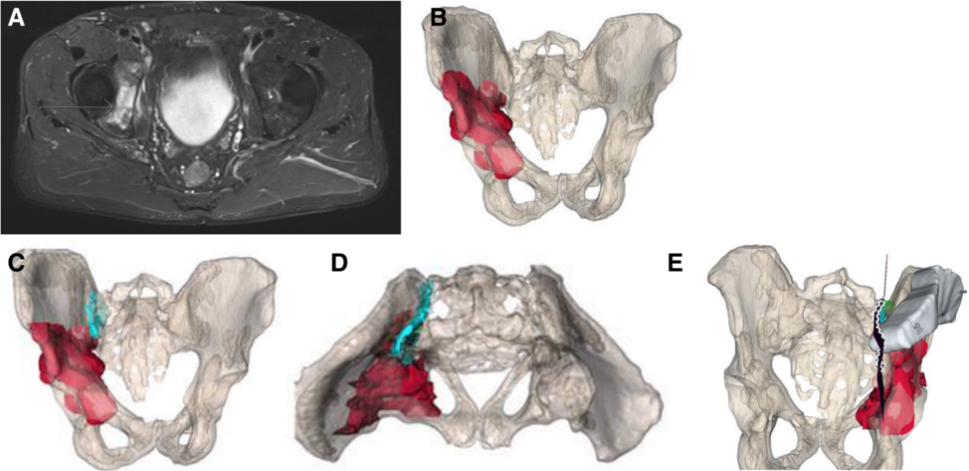
Case 4: preoperative planning. Due to close proximity of the tumor (*red*) to the sacroiliac joint (*cyan*), an osteotomy plane (*green*) medially to the joint, but laterally to the sacroiliac foramina was chosen. **a** Preoperative imaging of chondrosarcoma of the right acetabulum (*arrow*). **b** Preoperative planning. Antero-posterior view of the tumor localization in the pelvis. **c** Preoperative planning. Antero-posterior view of the tumor and sacroiliac joint. **d** Preoperative planning. Axial view of the tumor and sacroiliac joint. **e** A patient-specific instrument (PSI) is shown after placement onto the ilium

A 51-year-old male reported to his primary care physician with increasing pain around the right hip area for five months. The clinical findings did not show anything specific except for atrophy of the right quadriceps muscle. The patient was referred to another hospital, and an X-ray, MRI, and two biopsies were followed by radiofrequency decontamination of the biopsy tract to establish the diagnosis of chondrosarcoma of the right acetabulum without metastasis (Fig. 7). The tumor infiltrated the femoro-acetabular joint and penetrated the obturator muscle in the pelvis and sacroiliac joint.

**Fig. 8:**
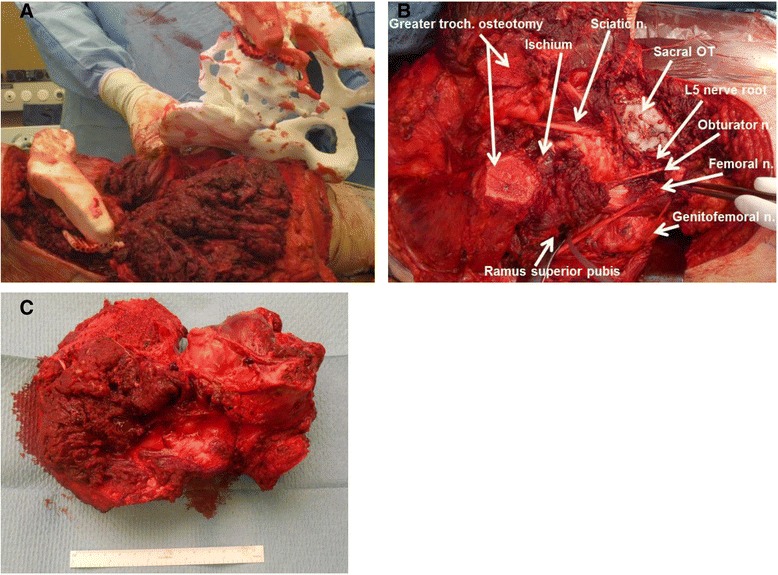
Case 4: intraoperative implementation of 3-D planning. **a** Placement of patient-specific instrument onto the patient (*bottom left*) and posterior view on the 3-D printed model with an identical patient-specific instrument (*upper right*). **b** Surgical field after resection. **c** Resection specimen

**Fig. 9:**
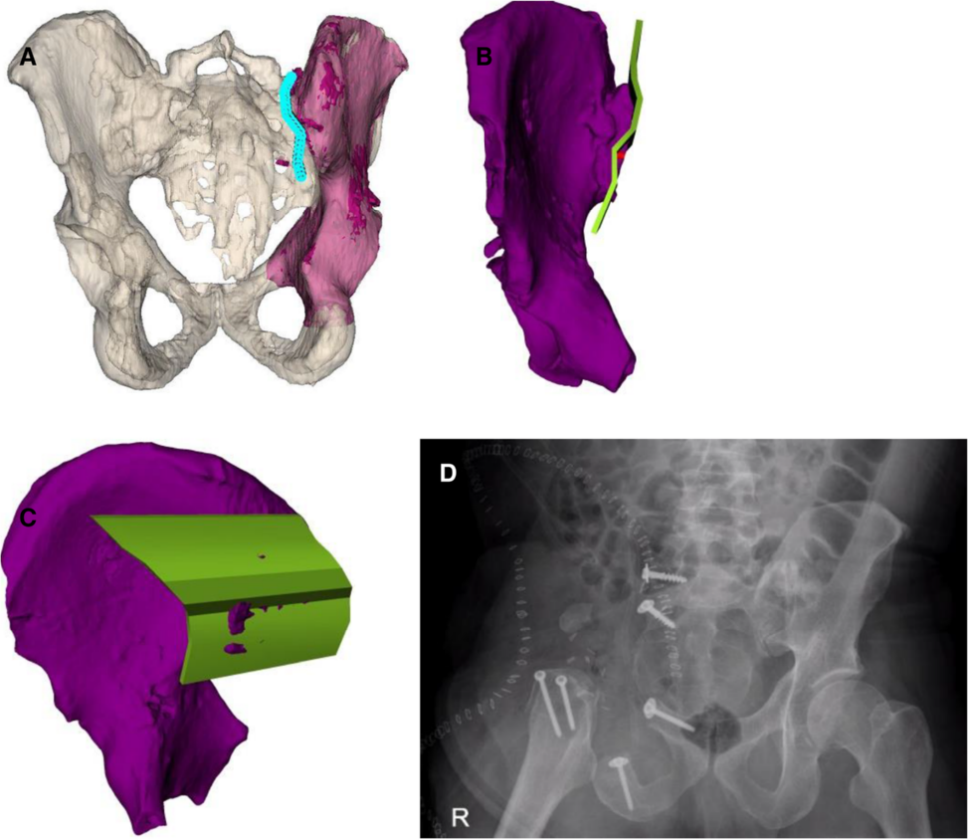
Case 4: intraoperative implementation of 3-D planning. Due to close proximity of the tumor (*red*) to the sacroiliac joint (*cyan*), an osteotomy plane (*green*) medially to the joint, but laterally to the sacroiliac foramina, was chosen to retrieve the resection specimen (*purple*). **a** Postoperative evaluation. Tumor resection specimen. **b** Postoperative evaluation. Anterior view of the tumor resection specimen with planned osteotomy plane and maximal error of 4 mm (*red*). **c** Postoperative evaluation. Posterior view of the tumor resection specimen with the planned osteotomy plane and maximal error of 4 mm (*red*). **d** Postoperative antero-posterior X-ray

Due to the vertical resection plane, no satisfactory prosthetic anchorage was possible. A hemipelvectomy including the femoral head (Enneking type I/II) and reconstruction with a modified Friedman Eilber procedure (Trevira tube, Implantcast GmbH, Buxtehude, Germany) were planned and performed [17, 18] (Figs. 7, 8, and 9). The freehand osteotomy planes were placed in the superior ramus of the pubic bone anteriorly, the supra-acetabular ischium inferiorly, while a cutting block was designed (in CASPA and manufactured by Medacta SA) to guide the osteotomy between the sacroiliac joint and the sacral foramina through the ala ossis sacri. The reconstruction between the pelvis and femur was carried out with Trevira tube, hinging on a second one connecting the pubis with the ischium.

Eleven days postoperatively, the patient developed severe headaches and homonymous hemianopsia toward the right side caused by subacute, ischemic infarction of the left cuneus. There were no other neurological sequelae, and he was treated with aspirin.

Histology revealed tumor-free margins. The patient was asked to refrain from hip movement and weight bearing for 3 months. Afterward, he was allowed to flex the hip up to 60° and weight-bear with 10 kilograms (kg). Six months postoperatively, the patient presented with beginning callus formation of painless non-dislocated insufficiency fractures of the sacrum and inferior pubic ramus. At the last follow-up, nine months postoperatively, no local recurrence or metastasis was observed and the insufficiency fracture of the sacrum had healed. He is currently flexing the hip up to 90° and weight-bearing with half of his body weight without any pain.

## Results

This study included four patients with pelvic sarcomas with a mean follow-up of 7.8 (range, 6.0–9.0) months (Table 1). Combining all available preoperative 3-D tools, i.e. 3-D planning, 3-D-printed models, and PSI with cutting blocks for osteotomies led to improved visualization and precision of tumor resection (maximal (max) error of 0.4 cm in case 4) compared to conventional methods using 3-D planning and freehand osteotomies (max error of 2.8 cm in case 1). The comparison of pre- and postoperative CT scans of case 1 revealed that the intraoperative entry points were not exactly chosen as planned resulting in malrotation of the resection specimen, which deviated from the superior entry and distal end points by 2.8 and 1.5 cm, respectively (Fig. 4). Altered measurements occurred due to ambiguous reference points caused by impeding soft tissue. Tumor-free margins were obtained in three patients (while one patient could not be evaluated due to curettage of a giant cell tumor). No signs of local recurrence or metastasis were observed. Postoperatively, two insufficiency fractures without substantial clinical consequences were seen.

## Discussion

This study illustrates different levels of 3-D planning and implementation in pelvic tumor surgery. It ranges from conventional planning and freehand osteotomies over the use of 3-D-printed models to PSI with cutting blocks. The first case demonstrates that the sole use of reference point-based measurements obtained from 3-D planning is rather inaccurate for intraoperative resection accuracy when the resection is performed in a freehanded fashion without PSI. Although this did not impact on tumor-free margins in the presented case, it may potentially lead to inadequate margins or removal of unnecessarily high amounts of healthy tissue in other cases. The second and third cases show that 3-D-printed models help visualize tumor dimensions and their proximity to vital structures much better. The sterilized 3-D-printed models can also be used to compare the intraoperative situation with the 3-D-printed models. The last case demonstrates that the additional use of a cutting block allowed a more precise pelvic tumor resection than the freehand osteotomies of our series, particularly in anatomically complex regions.

In the first case, which only used conventional 3-D planning executed by freehand osteotomies, it was surprising that the resection margin was closer to the tumor compared to what was planned preoperatively (Fig. 2). The postoperative CT analysis of the specimen demonstrated the pitfalls of freehand osteotomies after manual measurements of entry sites. These measurements and, in part, the subsequent osteotomy plane can differ from the preoperative planning due to impeding soft tissue. Although the tumor was removed completely due to the deliberate use of large margins, it may not have been removed en bloc if removal of the less healthy bone would have been opted for. Yet, this resulted in the removal of more healthy tissue than possibly needed. In the second and third cases, 3-D-printed models support the assessment of the tumor location and its surrounding structures such as nerve roots. They are also useful for repeatedly taking intraoperative measurements.The first three cases illustrate well how preoperative 3-D planning and visualization using patient-specific 3-D-printed models may deceive the surgeon and suggest a subjective precision that cannot be guaranteed. This is mainly due to difficulties in choosing the correct entry site for an osteotomy. In the fourth case, not only 3-D planning and 3-D-printed models were used but also cutting blocks were generated and utilized to improve the accuracy of the osteotomies. The comparison of pre- and postoperative margins showed a maximal deviation of planned resection of 0.4 cm.

The fact that PSI are associated with better precision than freehand osteotomies has been shown by Khan et al [14], who demonstrated an accuracy of 0.9 cm for freehand and 0.2 cm for PSI osteotomies on six matched pair cadaveric femurs. Although the accuracy for navigated and PSI osteotomies have been similar in a cadaveric study by Wong et al [19], PSI was significantly faster (16.2 versus 1.1 minutes (min), respectively). The presented precision with PSI of 0.4 cm is similar to a previous study by Vlachopoulos et al [11], who examined corrective osteotomies of the forearm in 14 patients and reported a precision of 0.2 cm. Above all, an osteotomy through the ala ossis sacri using cutting block assistance as opposed to freehand procedure alone provides great help for the surgeon. Furthermore, there are very few studies about the new technique of using PSI for pelvic tumor surgeries. A recent study by Gouin et al [15] reported that the mean location accuracy was 2.5 mm and the mean error on safe margin defined as the difference between the achieved and desired resection margins was −0.8 mm in nine cut planes of seven patients out of a case series of 11 patients with pelvic tumors. They reported a relatively high complication rate with five deep infections. Our study adds information about a comparison of PSI with freehand osteotomies and 3-D-printed models and detailed perioperative management. Another study by Cartiaux et al [13] demonstrated no difference in resection precision between junior and senior surgeons when using PSI on a synthetic pelvic bone model.

Despite the relatively unprecise tumor resection due to limited availability of 3-D planning and freehand resection in the first case, the outcome was still good. This can be explained by considering the large safety margins that were chosen. The fact that the tumor was located at an anatomic site without immediate weight-bearing or intra-articular function, or neurovascular bundles at risk, allowed for a larger error in resection precision. Had the tumor been located near structures at risk such as in case 4, resection with the presented error may have actually led to a different outcome. Furthermore, although the final outcome was good, three of four patients had complications. Asymptomatic insufficiency fractures of the inferior pubic ramus were observed in two patients. Both reported insufficiency fractures were not caused by the use of PSI but were more likely due to the nature of the disease and invasiveness or area of a tumor surgery. With advancing imaging techniques, i.e., more specific MRIs, complications such as insufficiency fractures may become more apparent albeit asymptomatic. Insufficiency fractures may possibly be reduced by thoroughly instructing patients about the importance of restricted weight-bearing with a maximum of 15 kg in the immediate postoperative phase for at least 6 weeks and a consecutive step-wise increase in weight-bearing. Moreover, patients need to be educated about this potential adverse effect when obtaining informed consent. Although insufficiency fractures at the pubic rami may not be prevented at all times due to altered biomechanics, they are usually managed successfully conservatively. Overall, tumor surgeries at the pelvis are difficult. Theoretically, any technique that allows more accuracy and precision, such as the presented one, may positively affect the overall outcome by only resecting diseased structures, therefore limiting unnecessary weakening of the bony structures, avoiding blood loss, and reducing morbidity.

The limitation of this study is the small number of patients with a heterogeneous group of sarcomas at the pelvis. Other potential drawbacks of 3-D planning and PSI are that surgeons need to make sure to account for impeding soft tissue. If PSI do not fit well, a false sense of certainness may arise. Therefore, it is important to realize if a cutting block cannot be positioned properly. This may certainly be associated with a learning curve. Moreover, custom-made 3-D-printed models and PSI are made for each particular individual, cannot be used for more than one patient, and may, therefore, be costly.

Overall, the use of 3-D printed models and, especially, PSI with cutting blocks in pelvic sarcoma surgery offered help to surgeons in our study. These tools provide more safety and accuracy that had previously been lacking with (3-D) planning without these tools. They also potentially offer better precision for tumor resection, which may in turn enable a surgeon to maintain a controlled distance to vital structures without having to compromise tumor-free resection margins and overall patient survival. This is crucial in a region such as the pelvis, where important anatomical structures are in close proximity to each other. Attempts to combine navigation systems and PSI seem promising and require more prospective studies [20].

## Conclusions

Three-dimensional planning as well as the intraoperative use of 3-D printed models and PSI is useful for sarcoma resection at the pelvis. Three-dimensionally printed models may help visualization and subjective precision. Patient-specific instruments with cutting blocks assist in performing precise osteotomies in order to obtain adequate resection margins.

## Abbreviations

ASIS: Anterior superior iliac spine
cm: Centimeters
CT: Computed tomography
S1: First sacral
G: Grade
kg: Kilograms
mm: Millimeters
MRI: Magnetic resonance imaging
Max: Maximal
M: Muscular strength
PSI: Patient-specific instruments
PET-CT: Positron emission tomography-computed tomography
PSIS: Posterior superior iliac spine
3-D: Three-dimensional
